# Community knowledge, practices, and dog management toward rabies in Hung Yen province, Vietnam: Insights for strengthening One Health interventions toward the 2030 zero-death goal

**DOI:** 10.14202/vetworld.2025.3509-3519

**Published:** 2025-11-27

**Authors:** Thi Thu Tra Vu, Thi Ngan Mai, Van Hieu Dong, Ha Thai Truong, Thi Thanh Tran, Harish Kumar Tiwari

**Affiliations:** 1 Department of Veterinary Public Health, Faculty of Veterinary Medicine, Vietnam National University of Agriculture, Hanoi, Vietnam; 2 Department of Veterinary Microbiology and Infectious Diseases, Faculty of Veterinary Medicine, Vietnam National University of Agriculture, Hanoi, Vietnam; 3 Pharmaceutical and Veterinary Material Company, Hung Yen, Vietnam; 4 Jyoti and Bhupat Mehta School of Health Science and Technology, Indian Institute of Technology, Guwahati, Assam, India; 5 Sydney Medical School, Faculty of Medicine and Health, University of Sydney, New South Wales, Australia

**Keywords:** dog management, Hung Yen, knowledge, One Health, practices, rabies, Vietnam

## Abstract

**Background and Aim:**

Rabies remains a fatal but preventable zoonotic disease causing nearly 59,000 human deaths annually worldwide, including approximately 75 cases/year in Vietnam. Despite the National Rabies Prevention and Control Program (2022–2030) targeting zero human deaths, community-level awareness and responsible dog management remain key determinants of program success. This study assessed knowledge, attitudes, and practices related to rabies prevention and dog management among residents of Hung Yen province.

**Materials and Methods:**

A community-based cross-sectional survey was conducted among 210 residents from Khoai Chau, Van Lam, and Yen My districts between November and December 2022. Data were collected using a structured, pre-tested questionnaire covering demographics, rabies knowledge, and dog ownership practices. Descriptive statistics and Chi-square tests were performed in R software, with p < 0.05 considered statistically significant.

**Results:**

Among 210 respondents, 82.4% had heard of rabies, and 60.7% demonstrated good knowledge. Most knew that dog bites transmit rabies (97.7%), but 39.9% were unaware that scratches can also cause infection. More than half (52.6%) still believed traditional remedies could prevent rabies, and 39.7% did not seek medical care after dog bites. Younger respondents (18–40 years) had significantly better knowledge (odds ratio = 1.90; p = 0.04). Among 85 dog owners, 82.4% vaccinated their dogs, yet 21.2% allowed dogs to roam freely, and 91.8% did not spay or castrate them. Higher socioeconomic status and urban residence were associated with better dog management practices.

**Conclusion:**

Despite Hung Yen’s low rabies fatality, notable gaps persist in community awareness and responsible pet ownership. Misconceptions about transmission routes and reliance on traditional treatment threaten progress toward the 2030 rabies-free goal. Strengthened One Health-based education, targeting older adults and low-income rural groups, is recommended to promote timely post-exposure prophylaxis and sustainable dog vaccination and population control programs.

## INTRODUCTION

Rabies is a fatal zoonotic disease that remains a major public health concern globally. It is responsible for an estimated 59,000 human deaths each year, with approximately 96% of these occurring in Asia and Africa [1]. The disease is caused by a neurotropic virus belonging to the genus *Lyssavirus* within the family *Rhabdoviridae* and is primarily transmitted through the bite of an infected animal through saliva [2, 3]. Among terrestrial mammals, domestic dogs serve as the principal reservoir and are responsible for more than 99% of reported human rabies cases worldwide [4]. Despite its lethality, rabies is entirely preventable through timely vaccination in both humans and animals [5–7]. However, the disease disproportionately affects poor and marginalized populations in developing countries due to limited awareness, cultural misconceptions, and inadequate preventive practices [8–10].

In Vietnam, 458 human rabies deaths were reported between 2011 and 2015, with most cases concentrated in the northern and central mountainous provinces [11]. To reduce this burden, the National Program for Rabies Control and Elimination (2017–2021) was launched, emphasizing key measures such as responsible dog ownership, vaccination of pet dogs, administration of post-exposure prophylaxis (PEP) to bite victims, outbreak investigation, public awareness campaigns, and strengthening of surveillance and diagnostic systems [11]. Nevertheless, human rabies deaths have remained relatively high, with 378 fatalities recorded between 2017 and 2021 [12]. In response, the Prime Minister of Vietnam approved the National Rabies Prevention and Control Program for 2022–2030 (Decision No. 2151/QD-TTg, dated December 21, 2021), which aims to achieve zero human deaths from rabies by 2030. This ambitious goal underscores the need for a strengthened One Health approach, involving coordinated action from the Ministry of Health, Ministry of Agriculture and Rural Development, and other governmental and international partners to enhance public awareness, dog population management, vaccination coverage, and disease surveillance [13].

Despite Vietnam’s ongoing national efforts to eliminate rabies, community-level understanding and preventive behaviors remain heterogeneous across provinces. Most previous knowledge, attitude, and practice (KAP) studies on rabies in Vietnam have focused on high-incidence provinces such as the northern midland and mountainous regions, where human fatalities remain frequent [14, 15]. However, limited attention has been given to provinces with low or no recent human deaths but that remain epidemiologically vulnerable due to their proximity to high-risk areas, dense human–animal interfaces, and increasing dog populations.

Hung Yen province, located in the Red River Delta and adjacent to Hanoi, represents a low-fatality yet at-risk region that has reported no human rabies deaths between 2017 and 2021, despite having extensive dog ownership and unrestricted dog movement. In such settings, the absence of fatalities can lead to complacency and a decline in participation in vaccination and public health interventions. Furthermore, there is a paucity of evidence on how socio-demographic variables, such as age, education, socioeconomic status, and place of residence, influence the community’s knowledge and practices regarding rabies prevention and dog management in these low-burden regions.

This knowledge gap limits the ability of health authorities and the One Health network to design context-specific education and intervention strategies. Therefore, a localized assessment of rabies-related knowledge, risk perception, and dog management practices is essential to inform targeted communication campaigns and support Vietnam’s 2030 zero-death goal.

The present study aimed to evaluate the level of community KAP related to rabies prevention and dog management among residents of Hung Yen province, Vietnam. Specifically, the study sought to:

1. Assess the extent of public knowledge regarding rabies transmission, prevention, and post-exposure behavior
2. Identify gaps and misconceptions influencing risky practices such as reliance on traditional remedies or delayed medical care
3. Examine associations between socio-demographic characteristics and rabies-related knowledge; and
4. Analyze dog ownership and management practices, including vaccination, confinement, and sterilization.

By generating baseline data from a low-fatality province, this study provides critical insights for refining local and national rabies control strategies under the One Health framework and supports the evidence base for Vietnam’s 2022–2030 National Rabies Prevention and Control Program.

## MATERIALS AND METHODS

### Ethical approval

Ethical approval for this study was obtained from the Institutional Review Board of the Institute of Malariology, Parasitology and Entomology Quy Nhon (Approval No. 720/VSR-LSĐT). All participants were informed about the objectives and procedures of the study prior to inclusion. Written or oral informed consent was obtained from each respondent, and all information collected was treated as confidential and used solely for research purposes.

### Study period and location

**Figure 1:**
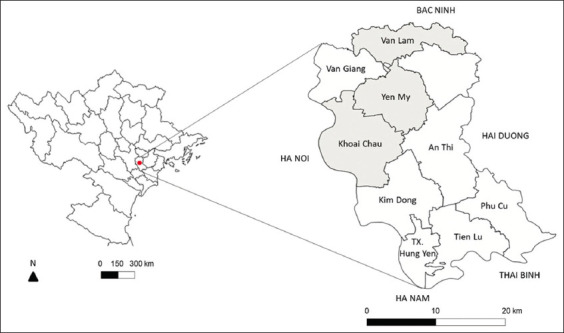
Study area: Hung Yen province in northern Vietnam (red circle) and Khoai Chau, Van Lam, and Yen My districts in Hung Yen (gray color). [Source: https://d-maps.com/carte.php?num_car=22381&lang=en].

Data collection was carried out over a 2-month period, from November to December 2022. The study was conducted in Hung Yen province, located in the Red River Delta region of northern Vietnam, adjacent to Hanoi, the national capital (Figure 1). The province covers an area of 930.2 km^2^ and comprises 10 administrative districts and cities (https://hungyen.gov.vn). Three districts, Khoai Chau, Van Lam, and Yen My, were randomly selected as representative study sites.

### Study design and sample size determination

This was a cross-sectional, community-based study designed to assess community knowledge and practices regarding rabies and dog management. The sample size was calculated using the formula n = Z² × P × (1–P)/d², assuming that 50% of the population in the three selected districts was aware of rabies, with a 95% confidence level and a 7% margin of error. Based on the total population of 483,982 residents (https://hungyen.gov.vn), the minimum required sample size was estimated to be 196 using Epitools (http://epitools.ausvet.com.au). To increase precision, 210 respondents were finally included in the study.

### Questionnaire design and sampling procedures

A structured questionnaire was developed to evaluate respondents’ demographic characteristics, knowledge about rabies, and dog ownership and management practices. The instrument consisted of three main sections:

1. Demographic information of the respondents
2. Knowledge assessment, comprising 19 questions on rabies transmission, prevention, and post-exposure practices, plus one question regarding responses to dog bites
3. Dog ownership and management practices, comprising 13 questions related to vaccination, confinement, and sterilization.

The questionnaire was pre-tested in a pilot study to ensure clarity and reliability and was revised accordingly. Due to the unavailability of detailed household lists in each selected district, convenience sampling was employed to recruit participants.

### Data collection

Data were collected through face-to-face interviews conducted by trained enumerators. Interviews were administered to household heads; in their absence, another adult household member (≥18 years) was invited to participate. Respondents who reported having never heard of rabies were excluded from the knowledge assessment component of the analysis.

### Variables and scoring criteria

Socioeconomic status was classified into low, middle, or high categories based on respondents’ education and occupation, as described by Tiwari *et al*. [9]. For regression analysis, the middle and high categories were combined into a single high/middle category. Respondents’ knowledge of rabies was scored according to correct responses to knowledge-related questions. The median score was used as a threshold to categorize respondents into two groups:

- Good knowledge (score ≥ median)
- Inadequate knowledge (score < median).

Demographic variables, such as age and family size, were dichotomized based on their medians. All data were entered into Microsoft Excel 2021 (Microsoft Office, Washington, USA) and analyzed using R Statistical Software version 3.4.3 (R Core Team, Vienna, Austria).

### Statistical analysis

All explanatory variables were converted into binary categorical variables. The association between demographic or socioeconomic factors and knowledge level was assessed using the Chi-square test. The odds ratio (OR) and 95% confidence interval (CI) were calculated to determine the strength of association between explanatory and outcome variables. Variables with a p ≤ 0.25 in bivariate analysis were included in the multivariate logistic regression model, using backward stepwise selection to control for confounding factors [16]. Only variables with p < 0.05 were retained in the final model.

## RESULTS

### Demographic characteristics of respondents

A total of 210 respondents were interviewed for this study. The demographic profile of the respondents is summarized in Table 1. The majority were female (60.5%), aged 40 years or older (52.9%), and from a low socioeconomic group (54.3%). Most respondents resided in communes (80.0%) and lived in households with four or fewer members (61.0%).

**Table 1:** Demographic characteristics of the respondents.

Variable	Total number of respondents (n = 210)		Respondents heard about rabies (n = 173)	
	
	Frequency (n)	Percentage	Frequency (n)	Percentage
Gender				
Male	83	39.5	76	43.9
Female	127	60.5	97	56.1
Age (years)				
18–40	99	47.1	88	50.9
>40	111	52.9	85	49.1
Socio-economic status				
Low	114	54.3	85	49.1
Middle/high	96	45.7	88	50.9
Residence				
Commune	168	80.0	134	77.5
Town	42	20.0	39	22.5
Family size				
≤4	128	61.0	106	61.3
>4	82	39.0	67	38.7
Own pets				
Yes	103	49.0	86	49.7
No	107	51.0	87	50.3
Pet species				
Dog	85	40.5	71	41.0
Cat	30	14.3	26	15.0
Other*	9	4.3	8	4.6

* Bird, chicken, and fish.

### Awareness and sources of information about rabies

Among the 210 respondents, 82.4% (173/210) reported having heard about rabies. The primary source of rabies information was television (60.0%), followed by public loudspeaker announcements (35.2%) and radio broadcasts (22.4%). The median family size among respondents was four, and the median knowledge score was 13. Based on the scoring system, 60.7% (95% CI: 53.0%–67.9%) of respondents who were aware of rabies demonstrated good knowledge of the disease (Table 2).

**Table 2:** Distribution of respondents’ knowledge of rabies (n = 173).

Criteria	Frequency (n)	Percentage
Know the agent that causes rabies		
Yes	89	51.5
No	84	48.5
Know that domestic and wild animals and humans are the targets of rabies		
Yes	44	25.4
No	129	74.6
Knowledge of the signs and symptoms of rabies in animals		
Yes	7	4.1
No	166	95.9
Dogs can transmit rabies to humans		
Yes	169	97.7
No	4	2.3
Cats can transmit rabies to humans		
Yes	153	88.4
No	20	11.6
Other animals can transmit rabies to humans		
Yes	94	54.3
No	79	45.7
Dog is the most common cause of rabies in humans		
Yes	155	89.6
No	18	10.4
Animal bites can transmit rabies to humans		
Yes	165	95.4
No	8	4.6
Scratches from animals can transmit rabies to humans		
Yes	104	60.1
No	69	39.9
<15 years of age is the group at greatest risk of rabies		
Yes	46	26.6
No	127	73.4
Rabies is a dangerous disease		
Yes	169	97.7
No	4	2.3
Rabies can be fatal		
Yes	165	95.4
No	8	4.6
Rabies can be prevented		
Yes	170	98.3
No	3	1.7
Know that traditional treatments cannot prevent rabies		
Yes	82	47.4
No	91	52.6
Know what should be done immediately after an animal bite		
Yes	161	93.1
No	12	6.9
Know how to wash wounds after an animal bite		
Yes	125	72.3
No	48	27.7
It is necessary to go to the health station/hospital if an animal bites people		
Yes	150	86.7
No	23	13.3
Post-bite anti-rabies vaccine can prevent human rabies		
Yes	137	79.2
No	36	20.8
Post-bite anti-rabies vaccine injection can cause health problems		
Yes	84	48.6
No	89	51.4

### Knowledge and factors associated with rabies awareness

Bivariate analysis indicated that several demographic factors were significantly associated with rabies knowledge. Male respondents were more likely to know that all animals could be affected by rabies (OR: 2.28, 95% CI: 1.14–4.58; p = 0.019) and that traditional treatments could not prevent the disease (OR: 2.35, 95% CI: 1.27–4.35; p = 0.006). Similarly, younger respondents aged 18–40 years were significantly more knowledgeable about the need to seek medical care after an animal bite (OR: 2.68, 95% CI: 1.04–6.90; p = 0.035) and the preventive value of post-bite vaccination (OR: 2.92, 95% CI: 1.33–6.40; p = 0.006).

Respondents from middle/high socioeconomic groups also demonstrated better awareness of rabies infection targets (OR: 2.62, 95% CI: 1.27–5.41; p = 0.008). A summary of associations between demographic characteristics and knowledge levels is presented in Table 3.

**Table 3:** Association between respondents’ knowledge of rabies and descriptive variables.

Variable	No. of good knowledge	OR (95% CI)	p-value (Chi-square test)
Gender			
Female (97)	55	1	
Male (76)	50	1.47 (0.79–2.73)	0.224*
Age (year)			
>40 (85)	45	1	
18–40 (88)	60	1.90 (1.03–3.54)	0.04*
Socio-economic status			
Low (85)	47	1	
Middle/high (88)	58	1.56 (0.85–2.89)	0.153*
Residence			
Commune (134)	79	1	
Town (39)	26	1.39 (0.66–2.95)	0.386
Family size			
>4 (67)	38	1	
≤4 (106)	67	1.31 (0.70–2.45)	0.394
Pet ownership			
No (87)	56	1	
Yes (86)	49	0.73 (0.40–1.35)	0.320
Dog ownership			
No (102)	65	1	
Yes (71)	40	0.73 (0.40–1.36)	0.328
Cat ownership			
No (147)	88	1	
Yes (26)	17	1.27 (0.53–3.03)	0.595

CI = Confidence interval, OR = Odds ratio,

* p < 0.25. These variables were evaluated using multivariate analyses.

Of the eight variables analyzed, three, gender, age, and socioeconomic status, showed associations with knowledge at a significance threshold of p < 0.25 and were included in the multivariate model. However, no significant predictors were retained after backward stepwise logistic regression.

### Post-exposure practices among dog-bite victims

Out of 63 respondents who reported being bitten by a dog, 52.4% (95% CI: 39.5%–65.0%) cleaned the wound immediately, and 60.3% (95% CI: 47.2%–72.2%) sought medical attention or received post-exposure anti-rabies vaccination. Meanwhile, 38.1% (95% CI: 26.4%–51.2%) monitored the biting dog for 10 days, and a small proportion (4.8%; 95% CI: 1.2%–14.2%) applied traditional remedies instead of medical treatment.

### Dog ownership and management practices

Among the 85 respondents who owned dogs, 82.4% (95% CI: 72.2%–89.5%) vaccinated their pets against rabies (Table 4). Most owners (69.4%; 95% CI: 58.3%–78.7%) kept their dogs within the household premises, and 76.5% (95% CI: 65.8%–84.7%) provided cages for confinement. However, 21.2% (95% CI: 13.4%–31.7%) allowed their dogs to roam freely, while 31.8% and 78.8% did not use leashes or muzzles, respectively. Only 8.2% (95% CI: 3.7%–16.8%) of owners reported having their dogs spayed or castrated.

**Table 4:** Dog management practices according to owner demographics (n = 85).

Variable	Frequency (n)	Percentage
Total number of dogs		
1	52	61.2
2	20	23.5
≥3	13	15.3
Breed of the dogs		
Domestic	50	58.8
Exotic	28	33.0
Both	7	8.2
Source of the dogs		
Purchased	74	87.1
Gifted	11	12.9
Registering for dogs		
Yes	17	20.0
No	68	80.0
Take the dogs to a veterinarian		
Yes	31	36.5
No	54	63.5
Rabies vaccination for dogs		
Yes	70	82.4
No/not remember	15	17.6
Time of the last rabies vaccine administration (n = 70)		
Within this year	44	62.8
Last year	13	18.6
Not remembering/>2 years	13	18.6
Let the dog roam free outside		
Yes	18	21.2
No	67	78.8
Always keep your dogs at home		
Yes	59	69.4
No	26	30.6
Dog cage		
Yes	65	76.5
No	20	23.5
Use leashes		
Yes	58	68.2
No	27	31.8
Use muzzles		
Yes	18	21.2
No	67	78.8
Spaying/castration		
Yes	7	8.2
No	78	91.8

### Socioeconomic factors influencing dog management

Bivariate analysis revealed significant associations between socioeconomic characteristics and responsible dog ownership practices. Respondents in the middle/high socioeconomic group were more likely to register their dogs (OR: 3.20, 95% CI: 1.07–9.55; p = 0.032), maintain cages (OR: 4.29, 95% CI: 1.14–16.08; p = 0.023), and spay or castrate their dogs (OR: 12.72, 95% CI: 1.45–111.38; p = 0.016).

In addition, respondents living in towns were more likely than those in communes to seek veterinary care for their dogs (OR: 4.96, 95% CI: 1.18–20.86; p = 0.046). Overall, better socioeconomic standing and urban residence were positively correlated with improved rabies prevention and dog management behaviors.

## DISCUSSION

### Overview of key findings

This study assessed community knowledge and practices regarding rabies and dog management in Hung Yen province, one of nine provinces in Vietnam that reported no human rabies deaths between 2017 and 2021 [12]. Despite the absence of fatalities, 82.4% of respondents had heard about rabies, which was lower than levels reported in India (96.4%), Indonesia (90.9%), and Cambodia (99.4%) [9, 17, 18]. These findings suggest that awareness of rabies remains suboptimal even in low-fatality but at-risk regions, underscoring the need for sustained education and communication efforts.

### Demographic influence on rabies knowledge

Age was a significant determinant of knowledge about rabies. Adults aged 18–40 years were more likely to have adequate knowledge than older respondents (>40 years). This result is consistent with a study in Indonesia [17] but contrasts with findings from the Amhara region of Ethiopia, where individuals aged 31–45 years demonstrated better awareness [16]. The higher level of knowledge among younger respondents in Hung Yen may reflect greater access to information through education, digital media, and public campaigns.

Although gender and socioeconomic status showed associations with rabies knowledge in the univariate analysis, these factors were not retained in the final multivariate model. This suggests that while these variables may influence awareness, their effects are likely mediated by other factors such as education and information exposure.

### Knowledge gaps and misconceptions

Most respondents recognized that rabies is fatal and transmissible through animal bites; however, only 4.1% could identify clinical signs of rabies in animals, and just 25.4% knew that all domestic animals, wildlife, and humans can contract the disease. Alarmingly, 39.9% were unaware that scratches can transmit rabies, and 52.6% still believed or were unsure whether traditional remedies could prevent infection. These misconceptions highlight persistent cultural and informational barriers to effective rabies prevention.

Furthermore, 39.7% of respondents did not seek medical care following a dog bite, mirroring trends reported in Pakistan [19] and Indonesia [20]. Similar to other low- and middle-income settings, barriers such as the distance to health facilities, unavailability or cost of vaccines, and loss of income during treatment contribute to poor health-seeking behavior [21, 22]. Reliance on traditional healers remains common because they are perceived as more accessible and affordable [23, 24].

### Determinants of rabies knowledge and practices

Several previous studies have identified demographic and experiential variables that influence rabies knowledge and attitudes [16, 25, 26]. In the Philippines, personal experience with rabies was the strongest predictor of good knowledge and preventive behavior [25]. Similarly, a smaller household size was linked with better awareness in Burkina Faso [26]. However, in the present study, family size was not found to be associated with knowledge (p > 0.05). Despite including multiple variables, gender, age, socioeconomic status, residence, family size, occupation, and education, the multivariate model did not yield significant predictors, suggesting that knowledge determinants may vary contextually across populations.

### Dog vaccination and ownership practices

Dog vaccination coverage among owners in Hung Yen (82.4%) exceeded the ≥70% threshold recommended by the World Health Organization and adopted by the Vietnam National Rabies Prevention and Control Program (2022–2030) [27]. This rate was substantially higher than those reported in Cambodia (0.8%) and Indonesia (53.9%) [20, 28] but still requires sustained reinforcement to maintain herd immunity. In contrast, Laos reported only 13% coverage among owned dogs [29].

Factors such as owners’ knowledge, accessibility of vaccination services, and willingness to vaccinate influence coverage rates [29, 30]. In this study, dog owners with strong rabies knowledge were significantly more likely to vaccinate their pets (OR 4.48, 95% CI 1.16–17.92; p = 0.021), underscoring the role of education in promoting preventive behavior.

### Responsible dog ownership and management

Beyond vaccination, responsible dog ownership is essential for sustainable rabies control. The study showed that owners with better rabies knowledge were more likely to adopt good management practices, such as registering dogs (OR 4.94, 95% CI 1.45–16.74; p = 0.007), seeking veterinary care (OR 2.49, 95% CI 1.01–6.16; p = 0.046), restricting roaming (OR 6.17, 95% CI 1.63–23.31; p = 0.004), and keeping dogs at home (OR 3.45, 95% CI 1.26–9.44; p = 0.014).

Despite these positive trends, 91.8% of dog owners had not spayed or castrated their dogs, and 21.2% allowed their dogs to roam freely. Such practices contribute to uncontrolled dog populations and increase the risk of rabies transmission. The findings align with global evidence that behavioral interventions targeting responsible ownership complement vaccination efforts in breaking the dog-mediated transmission cycle [31, 32].

### Novelty and public health implications

This study is among the first to evaluate rabies-related knowledge and dog management practices in a low-fatality province of Vietnam. The results demonstrate that even in areas reporting no human deaths, substantial knowledge gaps and risky behaviors persist. These findings underscore the importance of ongoing community engagement, educational outreach, and One Health-driven interventions that integrate veterinary and human health services. Tailored strategies should focus on older adults, rural residents, and low-income groups to reinforce timely post-exposure care and responsible pet ownership.

## CONCLUSION

This study revealed notable gaps in community knowledge and practices regarding rabies prevention and dog management in Hung Yen province, a low-fatality but high-risk region of Vietnam. Although 82.4% of respondents had heard of rabies and 60.7% demonstrated good knowledge, substantial misconceptions persisted. Nearly 40% of respondents were unaware that scratches could transmit rabies, and more than half still believed or were uncertain that traditional treatments could prevent infection. Among dog-bite victims, only 60.3% sought medical care, reflecting gaps in health-seeking behavior. Encouragingly, 82.4% of dog owners vaccinated their pets, surpassing the ≥70% target recommended for rabies control, yet only 8.2% practiced spaying or castration, and 21.2% allowed dogs to roam freely. Age was the most significant factor influencing knowledge, with younger respondents (18–40 years) showing greater awareness than older individuals.

The findings highlight the need for continuous and targeted public education campaigns to correct misconceptions about rabies transmission, promote timely PEP, and enhance responsible dog ownership. Mass communication tools such as television and community loudspeakers, identified as primary information sources, should be leveraged more effectively. Strengthened collaboration between the veterinary and human health sectors, in alignment with the One Health approach, can ensure integrated surveillance, vaccination coverage, and behavior change communication, directly contributing to Vietnam’s 2030 zero-death target.

A major strength of this study lies in its focus on a province with no recent human rabies deaths, offering insights into latent behavioral risks that persist even in apparently low-burden settings. The study also provides valuable baseline data for policymakers and field implementers to evaluate community readiness for rabies elimination under the National Rabies Prevention and Control Program (2022–2030). However, it was limited to three districts in Hung Yen province and had a relatively small sample size (n = 210), which may not represent the entire province. Relying on self-reported data introduces recall bias, particularly concerning past bites and vaccination history, while social desirability bias may have led to overreporting of favorable practices.

Future research should expand to other provinces with varying rabies incidence levels to explore regional differences in awareness, health-seeking behavior, and compliance with vaccination programs. Longitudinal and mixed-method studies are recommended to evaluate the effectiveness of ongoing communication campaigns and to track behavioral changes over time. Overall, the study underscores that achieving rabies elimination in Vietnam will require not only sustained vaccination programs, but also community-driven behavioral transformation supported by intersectoral coordination. Bridging knowledge gaps, reinforcing responsible dog ownership, and embedding rabies prevention within a holistic One Health framework will be pivotal to achieving a rabies-free Vietnam by 2030.

## DATA AVAILABILITY

The supplementary data can be made available from the corresponding author on request.

## AUTHORS’ CONTRIBUTIONS

TTTV and HKT: Conceived and designed the study. VHD and TTT: Collected the data. TTTV, TNM, and HTT: Analyzed the data and interpreted the results. TTTV: Drafted the manuscript. All authors have read and approved the final version of the manuscript.
